# Molecular genetic and physical analysis of gas vesicles in buoyant enterobacteria

**DOI:** 10.1111/1462-2920.13203

**Published:** 2016-02-15

**Authors:** Yosuke Tashiro, Rita E. Monson, Joshua P. Ramsay, George P. C. Salmond

**Affiliations:** ^1^ Department of BiochemistryUniversity of CambridgeCambridgeCB2 1QWUK; ^2^ Applied Chemistry and Biochemical Engineering CourseDepartment of EngineeringGraduate School of Integrated Science and TechnologyShizuoka UniversityHamamatsu432‐8561Japan; ^3^ Curtin Health Innovation Research Institute Biosciences PrecinctFaculty of Health SciencesCurtin UniversityBentleyWA6102Australia

## Abstract

Different modes of bacterial taxis play important roles in environmental adaptation, survival, colonization and dissemination of disease. One mode of taxis is flotation due to the production of gas vesicles. Gas vesicles are proteinaceous intracellular organelles, permeable only to gas, that enable flotation in aquatic niches. Gene clusters for gas vesicle biosynthesis are partially conserved in various archaea, cyanobacteria, and some proteobacteria, such as the enterobacterium, *S* *erratia* sp. ATCC 39006 (S39006). Here we present the first systematic analysis of the genes required to produce gas vesicles in S39006, identifying how this differs from the archaeon *H* *alobacterium salinarum*. We define 11 proteins essential for gas vesicle production. Mutation of *gvpN* or *gvpV* produced small bicone gas vesicles, suggesting that the cognate proteins are involved in the morphogenetic assembly pathway from bicones to mature cylindrical forms. Using volumetric compression, gas vesicles were shown to comprise 17% of S39006 cells, whereas in *E* *scherichia coli* heterologously expressing the gas vesicle cluster in a deregulated environment, gas vesicles can occupy around half of cellular volume. Gas vesicle production in S39006 and *E* *. coli* was exploited to calculate the instantaneous turgor pressure within cultured bacterial cells; the first time this has been performed in either strain.

## Introduction

Bacteria can use multiple modes of taxis, including swimming, twitching, swarming and flotation. Flotation is enabled through the regulated production of intracellular buoyancy chambers: gas vesicles. Gas vesicle‐driven buoyancy facilitates the movement of photosynthetic cyanobacteria into water column positions, allowing exposure to wavelengths of light that can support phototrophy (Pfeifer, 2012). Gas vesicles can also increase bacterial surface area‐to‐volume ratios, and thereby help survival under environmental and nutritional stresses (Houwink, 1956; Walsby, 1972).

Gas vesicles are visible as light‐refracting structures under phase‐contrast microscopy (PCM), and bacterial colonies producing gas vesicles can be opaque on plates. All gas vesicles identified to date appear to be constructed from homologous proteins (Pfeifer, 2012). The primary gas vesicle structural protein, GvpA, assembles into tandem arrays that form ribs of the cylindrical vesicle (Englert and Pfeifer, 1993; Walsby, 1994). A second protein, GvpC, forms an exterior mesh on the gas vesicle surface, providing further structural support and influencing gas vesicle shape (Hayes *et al*., 1988; 1992; Walsby and Hayes, 1988; Englert and Pfeifer, 1993; Walsby, 1994; Offner *et al*., 2000). Recently, the structure of GvpF from the cyanobacterium, *Microcystis aeruginosa* was solved, and through electron microscopy, GvpF was shown to form a part of the gas vesicle structure (Xu *et al*., 2014). However, the precise biochemical roles of many other proteins found in gas vesicle gene clusters are unknown, although their stoichiometry is thought to be important (Shukla and DasSarma, 2004; Chu *et al*., 2011; Pfeifer, 2012).

Recently, we discovered gas vesicles in the enterobacterium, *Serratia* spp. ATCC39006 (S39006), a genetically tractable host (Ramsay *et al*., 2011). S39006 is a Gram‐negative bacillus that is pathogenic to plant and nematode hosts and produces two secondary metabolites: the tripyrrole red pigment, 2‐methyl‐3‐pentyl‐6‐methoxyprodigiosin (prodigiosin) and the β‐lactam antibiotic, 1‐carbapen‐2‐em‐3‐carboxylic acid (a carbapenem) (Coulthurst *et al*., 2005; Williamson *et al*., 2006). The genetic locus for gas vesicle biosynthesis in S39006 was determined, and gas vesicles were shown to be responsible for the opaque colony phenotype and buoyancy. Transcription of the gas vesicle gene cluster is cell density‐dependent [under the control of the SmaIR quorum‐sensing (QS) system], responsive to oxygen status, and controlled by the gas vesicle regulatory protein A, GvrA (a member of the NtrC family of regulators) encoded within the gas vesicle cluster (Ramsay *et al*., 2011; Ramsay and Salmond, 2012). Although there are obvious similarities between various gas vesicle gene clusters, the S39006 cluster does not encode the two widely studied regulatory proteins, GvpD and GvpE, that are found in *Halobacterium salinarum* strains and in *Haloferax mediterranei* (Englert *et al*., 1992; Ng *et al*., 2000; Zimmermann and Pfeifer, 2003; Hofacker *et al*., 2004). However, as in various organisms, the functionality of other proteins encoded by the gas vesicle genetic locus of S39006 is unknown.

Here, we describe a comprehensive analysis of the 16.6 kb gas vesicle cluster in S39006. In frame deletions were created for each of the 19 genes in the cluster and impacts on gas vesicle production investigated. Pressure nephelometry was used to determine the collapse pressure of gas vesicles, and volumetric occupancy of the bacterial cytoplasm by vesicles in S39006, and an engineered *E. coli*‐carrying gas vesicles, was assessed. We also exploited gas vesicles to calculate the instantaneous turgor pressure of both S39006 cells and the recombinant *E. coli* strain by examining the collapse pressure of gas vesicles under different growth conditions. This study presents a complete analysis of a single gas vesicle gene cluster and further demonstrates how gas vesicles can be exploited in *E. coli*, generating cells that are composed of ∼50% gas vesicles.

## Results

### The core gas vesicle gene composition in proteobacteria

**Figure 1:**
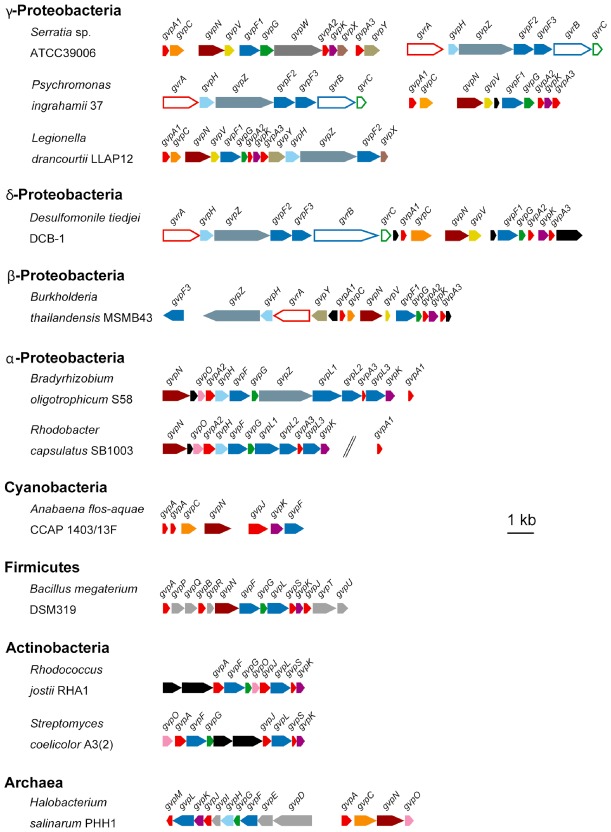
Organization of gas vesicle gene clusters. Gas vesicle gene clusters from the indicated organisms were annotated and compared. Genes of predicted similar function are denoted in the same colour. Genes predicted to encode gas vesicle regulatory proteins (Gvr proteins) are indicated as hollow arrows and gas vesicle proteins (Gvp proteins) are indicated as filled arrows. The scale bar indicates 1 kb.

S39006 carries a 16.6 kb cluster of 19 genes responsible for gas vesicle synthesis (Ramsay and Salmond, 2012). This gas vesicle gene composition is similar to that of other proteobacteria, particularly β‐, γ‐ and δ‐*proteobacteria*, possessing a gas vesicle gene cluster (Fig. 1). S39006 has three genes (*gvpA1*, *gvpA2* and *gvpA3*), encoding isoforms of the primary gas vesicle structural protein, GvpA; this is consistent with six proteobacteria that possess a gas vesicle gene cluster. A phylogenetic tree of GvpA showed that GvpA2 and GvpA3 are homologous to proteins previously annotated as GvpS and GvpJ, respectively, in other bacteria (Fig. S1 and Table S1). Despite differing annotations across organisms, all three GvpA variants contain a canonical GvpA domain. In addition, S39006 has three genes (*gvpF1*, *gvpF2* and *gvpF3*) encoding homologues of a reportedly minor structural protein, GvpF, and each is conserved in γ‐ and δ‐proteobacteria that carry a gas vesicle gene cluster (Fig. S1). Other genes encoding the gas vesicle‐associated proteins GvpC, GvpG, GvpH, GvpK and GvpN are also conserved in seven proteobacteria (Fig. 1 and Fig. S1). S39006 has five candidate gas vesicle proteins of unknown function: GvpV, GvpW, GvpX, GvpY and GvpZ, and of these, GvpV and GvpZ are conserved in four other β‐, γ‐ and δ‐proteobacteria gas vesicle gene clusters (Fig. 1).

### The gas vesicle locus in S39006 is defined by two distinct operons; one directly regulated by the QS morphogen signal, BHL

**Figure 2:**
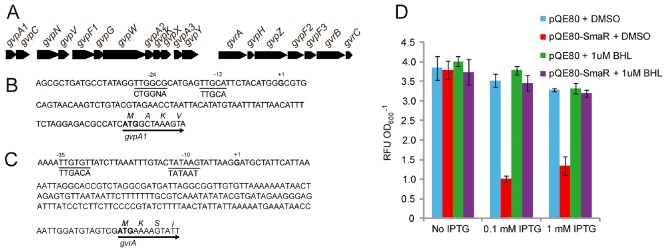
Operonic determination of S39006 gas vesicle cluster. A. Genetic organization of the S39006 gas vesicle gene cluster locus. B. The *gvpA* *1* transcriptional start site determined by 5′RACE is denoted in bold with a +1 above, and potential −12 and −24 sites of its promoter are underlined. Consensus sequences for σ^54^ are shown below. The translational start site is indicated with an arrow. C. The *gvrA* transcriptional start site, determined by 5′RACE, is denoted in bold with a +1 above, and potential −10 and −30 sites of its promoter are underlined. The consensus sequences for σ^70^ are shown below and the translational start site is indicated with an arrow. D. SmaR regulates *gvpA* *1* directly, but not *gvrA*. *E* *scherichia coli* strains carrying pRW50‐*gvpA* *1_pro_* were grown in the presence of either pQE80 or pQE80‐SmaR with added dimethylsulphoxide (DMSO) or 1 μM BHL dissolved in DMSO, where indicated. After 8 h of growth, samples were taken and β‐gal activity assayed (represented as RFU OD_600_^−1^). The values are the average of three biological replicates ± standard deviation (SD).

The 16.6 kb gas vesicle morphogenesis locus in S39006 is predicted to contain two operons, beginning with *gvpA1* and *gvrA* respectively. Reverse transcription polymerase chain reaction (PCR) and 5′‐RACE analysis showed that the two operons did not overlap and that there was no detectable read through between them (Fig. S2). The corresponding transcriptional start sites upstream of *gvpA1* and *gvrA* were determined (Fig. 2). Using reporter gene fusions, we showed previously that transcription of *gvpA1* is controlled by the σ^54^, NtrC‐like regulator GvrA. This is consistent with the location of a consensus σ^54^ binding site lying upstream of the *gvpA1* transcriptional start site.

The production of gas vesicles in S39006 is cell density dependent, controlled by the quorum‐sensing locus, SmaIR (Ramsay *et al*., 2011). SmaI produces the diffusible signalling molecule, *N*‐butanoyl‐L‐homoserine lactone (BHL) and, above a concentration threshold, BHL binds to SmaR, derepressing target gene expression (Slater *et al*., 2003). In a *smaI* mutant (BHL^−^), gas vesicles are absent but production can be restored by the addition of 1 μM BHL (Ramsay *et al*., 2011). BHL is therefore defined as a diffusible morphogen. To determine whether SmaR directly regulated expression of gas vesicles through either the *gvrA* or *gvpA1* operons, their respective promoters were first inserted upstream of a β‐galactosidase (β‐gal) reporter fusion. We investigated the expression of these two promoters in *E. coli* carrying either the *gvrA* or *gvpA1* promoter in the presence of a plasmid encoding SmaR, or with an empty vector control. In the presence or in the absence of SmaR, β‐gal expression from the *gvrA* promoter was the same (Fig. S2). In contrast, activity of the *gvpA1* promoter decreased by over 75% in the presence of SmaR, but this repression could be relieved by the addition of 1 μM BHL (Fig. 2). Together, these results suggest that QS‐dependent regulation of flotation in S39006 acts via direct transcriptional repression of *gvpA1*, but not *gvrA*.

### Comprehensive deletion analysis of the gas vesicle operons

**Figure 3:**
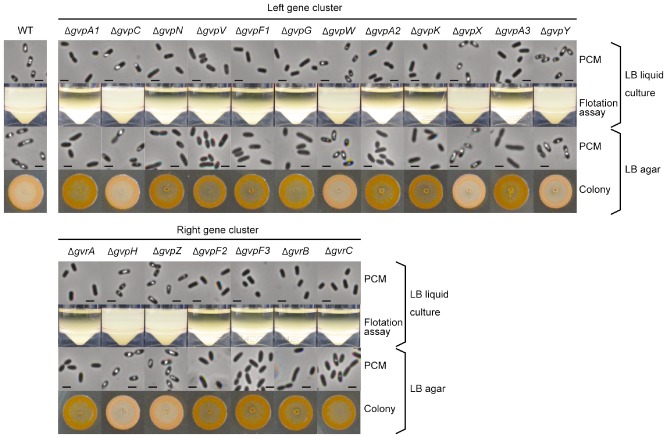
Comprehensive mutational analysis of the gas vesicle production locus. Gas vesicle production in the WT and each *gvp* or *gvr* mutant was assessed throughout both the *gvpA1* operon (top section) and *gvrA* operon (bottom section). Cells were grown in LB in sealed universals or on LB agar at 30°C for 24 h. Gas vesicle formation was confirmed by PCM observations from liquid culture (top row), flotation assays from liquid culture (second row), PCM from colonies grown on plates (third row) or colony morphology (fourth row). The scale bars in the PCM images represent 1 μm.

**Figure 4:**
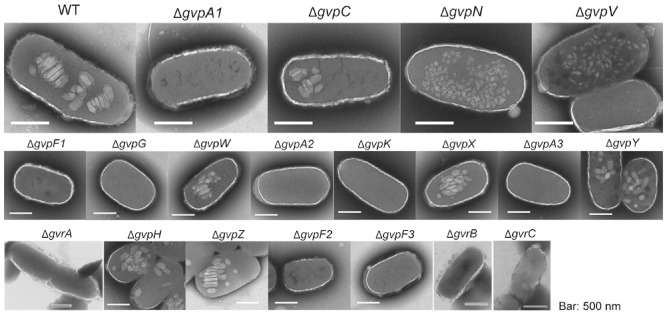
Transmission electron microscopy images of wild type (WT) and several *gvp* mutants. WT cells and mutants were grown in LB in sealed universals for 24 h and gas vesicles were observed by TEM (scale bars represent 500 nm).

Previous studies of the proteins required for construction of gas vesicles in archaea and cyanobacteria have focused largely on the main structural protein, GvpA, and the strengthening protein, GvpC (Walsby and Hayes, 1988; Walsby, 1994; Offner *et al*., 1996). In an earlier study on S39006, transposon insertion mutants and strains with reporter fusions in *gvpA1* and *gvrA* were analysed, but these insertion mutations were polar (Ramsay *et al*., 2011) so it was not possible to assess the impacts of the 19 individual *gvp* or *gvr* genes (mostly with no known function) on gas vesicle morphogenesis. Furthermore, as our bioinformatic analysis suggests, the core gene set found within gas vesicle clusters is likely to be widely conserved (Fig. 1). To elucidate the role of all 19 genes in the S39006 locus and to try to define the ‘minimum gene set’ required for gas vesicle morphogenesis, in frame deletions were constructed for each gene. Each mutant was scored on plates for the opaque colony morphotype (a facile indicator of gas vesicle presence); cultures were examined directly for phase‐bright vacuoles by PCM; and flotation assays were performed to determine if mutants were buoyant (Fig. 3). Transmission electron microscopy (TEM) images were also taken to observe the presence and shapes of gas vesicles (Fig. 4). From these data, we concluded that *gvpA1*, *F1*, *G*, *A2*, *K*, *A3*, *F2* and *F3* and *gvrA* and *B* were required for the formation of phase‐bright structures and for cell buoyancy (Fig. 3); none of the corresponding mutant strains produced gas vesicles detectable by TEM or by PCM under any growth conditions tested. In a *gvrC* mutant, a few gas vesicles were observed in some cells taken from a colony on a plate, but not in planktonic cells. Ectopic synthesis of the corresponding wild‐type proteins restored gas vesicle production in each deletant strain, confirming that each in‐frame knockout only affected one gene (Fig. S3). From these data, we concluded that the protein products of these 10 genes are each required for gas vesicle formation in S39006.

S39006 mutants defective in *gvpN* and *gvpV* did not float but contained small bicone‐shaped gas vesicles visible by TEM (Fig. 4). It has been observed in other bacteria that gas vesicles initially assemble in a bicone form before morphogenetic development into the full cylindrical form (for comprehensive reviews, see Walsby, 1994; Pfeifer, 2012). When wild‐type versions of either *gvpN* or *gvpV* were expressed in these S39006 mutants, *in trans*, gas vesicles were observed by PCM, and full‐size mature gas vesicles were seen by TEM (Fig. S4A). However, neither a deletion of *gvpN* nor *gvpV* could be complemented by excess of the other, suggesting that the two proteins perform independent functions (data not shown). Previous work demonstrated that applying pressure to wild‐type cells could lead to generation of similar bicone vesicles in S39006 and, in the absence of further stresses, these grew into the larger form (Ramsay *et al*., 2011). However, when we applied pressure to either a *gvpN* or *gvpV* mutant, bicone vesicles were still visible by TEM, suggesting that these bicone structures are robust (Fig. S4). The measurements of gas vesicles within *gvpN* or *gvpV* mutants showed that they were the same size in both strains, although significantly different from gas vesicles seen in the wild‐type strain (Fig. S4B). Taken together, these data suggest that GvpN and GvpV are essential for the morphogenetic assembly of gas vesicles from simple bicones into mature, functional gas vesicles in an S39006 developmental program.

There are three GvpA isoforms in S39006. Ramsay *et al* postulated that each of the GvpA proteins might relate to a different size of vesicle (2011). However, we found that loss of any one of the *gvpA* protein isoforms prevented gas vesicle formation (Fig. 3). Thus, we believe that all three GvpA isoforms seem to be essential for the production of mature gas vesicles in S39006.

### Robustness of gas vesicles under pressure

**Figure 5:**
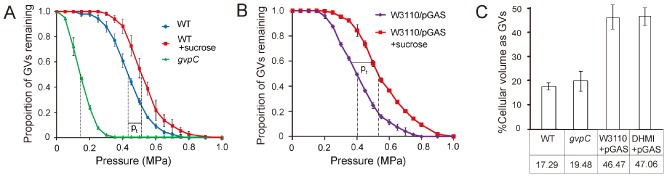
Physical characterization of gas vesicles and volumetric calculations in *E* *. coli* and S39006. (A) Collapse pressure experiments on S39006 or (B) *E* *. coli* carrying the pGAS cosmid. At 0.05 MPa pressure increments, the relative proportion of gas vesicles present in each sample was ascertained. The values are the average of three biological replicates ± SD. The black dashed lines indicate the mean collapse pressure, **p**_c_, for each sample and the continuous black line indicates **p**_t_, the turgor pressure. Where indicated, the collapse pressure was measured in cells exposed to LB medium containing 0.35 M sucrose. (C) Volumetric calculation of the percentage of gas vesicles in cells. Values indicated are the mean gas vesicle percentage of three biological replicates ± SD.

In other organisms, GvpC is thought to form a meshed structure on the surface of gas vesicles, providing additional strength (Walsby and Hayes, 1988; Hayes *et al*., 1992; Pfeifer, 2012). Gas vesicles are phase bright within cells, but when collapsed, they no longer refract light. By applying incremental pressure and examining the change in light refraction, the mean critical pressure (***p***_c_), when 50% of gas vesicle has collapsed, could be determined. The ***p***_c_ of gas vesicles in S39006 was 0.4334 MPa and, in a *gvpC* mutant, it decreased to 0.1435 MPa (Fig. 5A). This decrease in ***p***_c_ was counteracted by expression of *gvpC in trans* (Fig. S5A). It was formally possible that this change in collapse pressure could have been because the gas vesicles in a *gvpC* mutant were a different shape and size. However, no significant differences in gas vesicle sizes were observed between a *gvpC* mutant and wild type (Fig. S4) and so these data confirm that GvpC_39006_ strengthens gas vesicles.

Gas vesicles can be produced in *E. coli* strain W3110 carrying the S39006 gas vesicle cluster expressed from the cosmid, pGAS (Ramsay *et al*., 2011). We used this strain to compare the behaviour of gas vesicles in *E. coli* and S39006. The ***p***_c_ of gas vesicles in *E. coli* strain W3110 carrying pGAS was very similar to that of the gas vesicles in S39006 (0.4012 MPa in *E. coli* and 0.4334 MPa in S39006), suggesting structural similarity in both natural and engineered hosts (Fig. 5B).

### Gas vesicles as tools for measurement of instantaneous turgor pressure of *E* *. coli* and S39006

Cells maintain a cytoplasmic solute balance relative to their environment, and the difference between external and internal concentrations results in turgor pressure. Instantaneous measurements of turgor within cells have proved problematic, but a method for measuring turgor pressure within live cells containing gas vesicles exists (Holland and Walsby, 2009). By measuring the relative change in ***p***_c_ when cells are exposed to medium containing 0.35 M sucrose, altering the balance of solute concentration relative to the local environment, turgor pressure (***p*_t_**) in live cells can be assessed. However, until now, this has only been used in cyanobacteria and never in proteobacteria such as *E. coli* or S39006 (Holland and Walsby, 2009).

Based on TEM observations and collapse pressure profiles, gas vesicles in *E. coli* and S39006 were indistinguishable. Thus, we were able to exploit them to calculate corresponding instantaneous turgor pressures by pressure nephelometry. We first examined the change in ***p*** *_c_* in S39006 between cells in Lysogeny Broth – Lennox (LB) or cells exposed to LB + 0.35 M sucrose. The difference in collapse pressures between these two conditions (***p*** *_t_*) was 0.1036 MPa (Fig. 5A), suggesting that the turgor pressure in S39006 was around 0.1 MPa. Similar experiments testing the difference in ***p*** *_c_* in *E. coli* indicated that ***p*** *_t_* was 0.1423 MPa (Fig. 5B). Thus, we have been able to exploit the presence of gas vesicles to determine turgor pressure for the first time in enterobacteria such as *E. coli*.

### Transcription of *gvpA* *1* is independently regulated by GvrA, GvrB and GvrC

We showed previously that transcription of *gvpA1* was dependent on prior expression of the *gvrA‐gvrC* operon, but it was unknown which specific genes were involved in this regulation (Ramsay *et al*., 2011) To study this further, we combined in frame mutations in *gvrA*, *gvrB* or *gvrC* in S39006 with a *gvpA1::uidA* reporter fusion and analysed β‐glucuronidase (β‐gluc) activity in these strains throughout growth. It was reported previously that β‐gluc activity increased exponentially in stationary phase in a strain carrying a *gvpA1::uidA* fusion alone. However, a significant decrease in β‐gluc activity, reflecting *gvpA1* expression, was observed in strains carrying *gvrA*, *gvrB* or *gvrC* mutations (Fig. S6). Taken together, these results suggest that GvrA, GvrB and GvrC are required independently for full expression of the *gvpA1* operon and gas vesicle synthesis in S39006.

## Discussion

Different taxis systems enable bacterial dissemination between niches. The components that make up the apparatus required for swimming and twitching motility have been widely studied and their assembly pathways are well defined (Macnab, 2003; Busch and Waksman, 2012). Although the importance of gas vesicles in facilitating flotation is understood, many organisms that produce gas vesicles, such as the archaeon *H. salinarum*, contain gas vesicle genetic clusters that contain genes absent from S39006, for example *gvpI* and *gvpO*, and a different regulatory hierarchy, most notably well‐characterized regulators GvpD and GvpE (Kruger *et al*., 1998; Hofacker *et al*., 2004; Scheuch *et al*., 2008; Bleiholder *et al*., 2012; Marschaus and Pfeifer, 2012). Therefore, comprehensive studies of gas vesicles in *H. salinarum* or the cyanobacterium *Anabaena flos‐aquae* are not necessarily readily comparable with those in S39006 (Hayes and Powell, 1995; Offner *et al*., 1996; 1998; 2000; Li and Cannon, 1998; Hofacker *et al*., 2004). Furthermore, not all bacteria that possess a putative gas vesicle genetic cluster may actually produce gas vesicles. For example, the actinomycete, *Streptomyces coelicolor* contains a cryptic gas vesicle gene cluster, but no conditions have yet been identified where gas vesicles are formed, implying that gas vesicles may perform another, unidentified, function in this soil bacterium (van Keulen *et al*., 2005). An alternative explanation may be that the organism simply lacks one or more proteins that are essential for gas vesicle production. The gas vesicle gene cluster in *S. coelicolor* lacks *gvpN*, *gvpV*, *gvpF2*, *gvrA*, *gvrB* and *gvrC*, all genes required for full cylindrical gas vesicle formation in S39006. Further, the GvpA protein encoded in the *S. coelicolor* cluster differs significantly from other gas vesicle‐producing organisms studied so far (Fig. S1 and van Keulen *et al*., 2005). Although there is variation between the different gene clusters, the required gene set described here may provide a starting point for ‘turning on’ gas vesicles in *S. coelicolor* and other bacterial species.

Although not all gas vesicle gene clusters contain the same genes in an identical order, they follow a general pattern, at least within the *Proteobacteria*. The gene cluster is divided into two operons, one starting with *gvpA1*, as in S39006. The second operon almost always begins with *gvrA* and includes genes with known regulatory function such as *gvrB* and *gvrC*. However, in *Psychromonas ingrahamii* 37 and *Desulfomonile tiedjei* DCB‐1 the order of the operons has been inverted, and in *Burkholderia thailandensis* MSMB43 the two operons are divergently transcribed. Despite these clear differences, the arrangement of genes is largely similar across many bacterial species (Fig. 1).

Gas vesicles are produced by the enterobacterium, S39006, a highly genetically tractable strain (Ramsay *et al*., 2011). Furthermore, the gas vesicle genetic module from 39006 can be functionally reconstituted in *E. coli*, and so the roles of each S39006 gene involved in gas vesicle production in both natural and heterologous hosts can also be addressed. Here, we have performed the first full mutational analysis of the two operons that form the gas vesicle cluster in S39006 and determined the minimal gene set required for gas vesicle synthesis. We have ascertained further information about proteins such as GvpN and GvpV, potentially acting as chaperones, that facilitate formation of cylindrical vesicles from small bicones in S39006. In *H. salinarum* PHH1, the deletion of *gvpN* resulted in smaller bicone gas vesicles, but this study was unable to define the function of GvpN and the corresponding mutations could not be complemented (Offner *et al*., 2000). Furthermore, in *H. salinarum* NRC‐1, deletion of *gvpN* from the mini‐chromosome pNRC100 also led to the formation of smaller vesicles (DasSarma *et al*., 1994). However, neither of these gas vesicle gene clusters contains a copy of *gvpV*, similar to that found in S39006. GvpV homologues have been identified in only five proteobacterial species, suggesting that this protein may perform a function specific to these bacteria (Fig. 1). However, we have shown that both GvpN and GvpV act independently to extend the gas vesicle shape from bicone to the full cylindrical form. Although this study has clarified the function of some gas vesicle genes, there are also differences in functions between organisms. For example, *gvpH* is found in all six proteobacterial strains we analysed, but it is absent from *A. flos‐aquae* and *Bacillus megaterium* (Fig. 1). In the archaeon *H. salinarum* PHH1, *gvpH* is present and, as in S39006, its deletion is not detrimental to gas vesicle formation, yet in a *gvpH* deletion of *H. salinarum*, gas vesicles were less stable when isolated and could not be visualized by TEM (Offner *et al*., 2000). However, an earlier study demonstrated that an insertion in *gvpH* resulted in significantly reduced production of gas vesicles from the gene cluster on the mini‐chromosome pNRC100 in *H. salinarum* NRC‐1 (DasSarma *et al*., 1994). In contrast, in S39006, we found no change in gas vesicle shape or stability in a strain carrying a *gvpH* mutation.

From previous TEM studies, it appeared that differently sized vesicles were made by S39006 (Ramsay *et al*., 2011), but an extensive microscopy analysis in this study has not shown statistically robust evidence supporting this interpretation. Our results could suggest a finely tuned assembly process with few visually obvious intermediate steps, as almost all of our mutants were either gas vesicle negative or revealed gas vesicles with no discernible phenotypic difference from those of the wild type. These results echo those observed in other studies of gas vesicle genetic clusters from *H. salinarum* NRC‐1 and PHH1 and in *M. aeruginosa*, where very few intermediate assembly steps were identified (DasSarma *et al*., 1994; Offner *et al*., 2000; Mlouka *et al*., 2004).

It seems unlikely that the deletion of these ‘non‐essential’ genes has no impact whatsoever in gas vesicle biogenesis. For example, in these experiments, we cannot assess the kinetics of gas vesicle assembly because we only monitor the behaviour of cell populations. Thus, it is formally possible that some of these ‘non essential’ genes might play very subtle roles in modulating gas vesicle formation that cannot be detected in crude assays or seen by TEM. It is also possible that S39006 modulates the structure of gas vesicles under varying physiological conditions that were not investigated in this work.

In addition to defining the minimum gene set required for gas vesicle formation in S39006, we were also able to physically characterize these gas vesicles. The collapse pressure of gas vesicles in S39006 (0.4334 MPa) is similar to that found in the cyanobacterium, *Microcystis* sp. 8401 (0.468 MPa) (Holland and Walsby, 2009) but is very different than the collapse pressure of gas vesicles in *Microcystis* sp. BC 84/1 (0.77 MPa; Walsby and Bleything, 1988) or *H. salinarum* (0.09 MPa; Walsby, 1972). Furthermore, as in *Anabaena* and other systems, removal of GvpC caused more than a threefold decrease in the critical collapse pressure (Buchholz *et al*., 1993). Thus, it appears that the structural robustness function of GvpC is conserved in S39006, *Anabaena* and *Microcystis* sp. 8401. Similarly, the role of GvpA1 (or GvpA in other systems) appears to be the main structural protein. However, the role of GvpA2/A3 (or the corresponding proteins GvpJ/S in other systems) is still unclear. In S39006, we have annotated these three proteins as variants of GvpA because all three isoforms contain clear GvpA domains, despite the different annotations used in other organisms. Finally, and most importantly, we have demonstrated that all three of these GvpA variants are required for the morphogenesis of functional gas vesicles in S39006.

An ongoing challenge in the study of cellular turgor pressure has been the difficulty of ascertaining instantaneous turgor in a living culture without damaging the cells. For example, atomic force microscopy has been used to measure the deformability of the cellular membrane (Arnoldi *et al*., 2000; Deng *et al*., 2011). Although this technique is useful, it can only be done at a single‐cell level, which may not reflect the average turgor pressure across a bacterial culture. However, the biosynthesis of gas vesicles can be exploited to determine the turgor pressure across cells in a heterogeneous culture. As gas vesicles are opaque, their collapse results in a change in culture turbidity. By monitoring the changes in pressure collapse under different conditions (say one where turgor pressure has been removed by placing the cells in an isotonic solution), the turgor pressure across a culture can be calculated. As we can now reproduce this in *E. coli*, for the first time, the effect of particular mutations in solute transport pathways, on internal cellular pressure can be determined. Potentially, this has widespread applications and may have utility in other bacterial systems, wherever they are capable of producing gas vesicles.

This study significantly enhances our understanding of gas vesicles in S39006, and raises multiple questions. First, although some roles for GvpA and GvpC have been documented in other systems, important questions remain about the functionality of proteins GvpA2 and A3 (or GvpJ and S in other systems). All three isoforms of GvpA (A1, A2 and A3) in S39006 contain a putative ‘GvpA’ domain, and our mutational and complementation analyses have now proved that all three GvpA variants are essential for gas vesicle formation in 39006. More information is clearly required about the structures of all three GvpA variants, how they may perhaps dock together, and their precise morphogenetic roles in gas vesicle development.

This work with in frame mutants has also defined several new proteins as essential for gas vesicle formation in S39006. For example, what are the precise roles of GvrA, B and C in the regulation of gas vesicle synthesis? Each of the three proteins is essential for gas vesicle formation under the growth conditions tested here, and each independently regulates transcription of the *gvpA1* operon, although they could be integrating different environmental or physiological cues in the signal transduction pathway to gas vesicle development. The specific role of the three GvpF variants in gas vesicle assembly also remains unclear. GvpF is widely conserved across different gas vesicle gene clusters (Fig. 1), and the protein has been identified in two proteomic gas vesicle analyses (Shukla and DasSarma, 2004; Chu *et al*., 2010). A recent report described the structure of GvpF from *M. aeruginosa* and showed that it localized to the gas‐facing surface of gas vesicles, suggesting that this protein does play a structural role (Xu *et al*., 2014). However, the precise role of GvpF in the structure or assembly of gas vesicles has never been elucidated. It also remains unclear why S39006 contains three GvpF isoforms. An earlier hypothesis that each GvpF variant might pair with one of the three GvpA proteins (Ramsay and Salmond, 2012) now seems unlikely, but further study of the structures and functions of these essential GvpF variants is required to clarify their role(s).

In summary, although the production of gas vesicles was originally described in 1895, the specific role of each protein required for gas vesicle formation is still being dissected (DasSarma *et al*., 1994; Offner *et al*., 2000; Mlouka *et al*., 2004; Pfeifer, 2012; Tavlaridou *et al*., 2013; 2014; Xu *et al*., 2014). This work describes the minimal gene set required for gas vesicles in S39006 and demonstrates that gas vesicles formed in *E. coli* and S39006 are physically indistinguishable. Gas vesicles have potential applications in multiple fields, including in nanoparticles (DasSarma *et al*., 2014), as a contrast agent in ultrasound imaging (Shapiro *et al*., 2014a, 2014b), and for delivery of antigens as fusion proteins (DasSarma *et al*., 2014). In the synthetic biology, chemical factory, biotechnology and bioprocessing arenas, the gas vesicle cluster could be exploited in *E. coli*, or other organisms, to engineer cells that float or sink in a tightly controlled way – perhaps in response to specific synthetic chemical signals. Furthermore, through the use of pressure nephelometry, the exploitation of the gas vesicle cluster in *E. coli* can now allow quantifiable assessments of the impact of any mutation or environmental condition on the instantaneous turgor pressure of a whole culture.

## Experimental procedures

### Bacterial strains, plasmids and culture conditions

*Serratia* sp. ATCC39006 LacA was the wild‐type parental strain for all mutant strains constructed and used in this work. Bacterial strains and plasmids used in this study are listed in Table S2. Additional details of construction of strains and plasmids are found in the *Strain construction and plasmid cloning* section below. Oligonucleotides used are listed in Table S3. S39006 and *E. coli* were grown in LB (10 g tryptone l^−1^, 5 g NaCl l^−1^, 5 g yeast extract l^−1^) or LB agar (1.5%) at 30°C or 37°C, respectively, and supplemented with antibiotics where necessary.

### Strain construction and plasmid cloning

In frame mutants carrying deletions of each gene in the S39006 gas vesicle cluster were constructed by homologous recombination using derivatives of the suicide vector pKNG101 listed in Table S2. For construction of *gvpA1* mutant, fragments flanking the open reading frame were amplified with oligonucleotide pairs, gvpA1_5F_BamHI/gvpA1_5R_XhoI, and gvpA1_3F_XhoI/gvpA1_3R_SpeI (all oligonucleotides are listed in Table S3). These fragments were used to perform overlap extension PCR, creating a DNA fragment lacking the *gvpA1* open reading frame, but containing the flanking DNA. The resulting DNA fragment was digested with BamHI/SpeI and ligated into compatibly digested pKNG101 to yield pKNG101‐Δ*gvpA1*. The *Escherichia coli* β 2163 harbouring this plasmid was used as a donor for conjugation, and marker‐exchange mutagenesis was carried out. The confirmation of the gene deletion was by PCR and sequence analysis. The construction of other mutants was performed in the same way with restriction enzymes corresponding to their respective oligonucleotides.

For the construction of pQE80*oriT*‐*gvpA1*, the *gvpA1* gene was amplified from the S39006 chromosome with oligonucleotides gvpA1F‐EcoRI and gvpA1R‐HindIII (Table S3), digested with EcoRI/HindIII and cloned in pQE80*oriT* under control of the *lac* promoter. The plasmid‐producing GvpA1 without a His_6_ tag was introduced into *Serratia* by conjugation using *E. coli* β 2163 as a donor strain. The construction of other plasmids containing *gvp* or *gvr* gene was performed similarly but with restriction enzymes corresponding to their respective oligonucleotides.

The promoter fusions of *gvrA_pro_* and *gvpA1_pro_* were created by PCR using oligonucleotides oREM399/400 and oREM397/398 respectively. These fragments were digested with EcoRI/HindIII and ligated into compatibly digested pRW50 and used to transform *E. coli* strain DH5α, creating pRW50‐*gvpA1*_pro_ and pRW50‐*gvrA*_pro_. Each of these strains was transformed with either pQE80 or pQE80‐SmaR (Slater *et al*., 2003).

### RNA studies

The cells from cultures grown in LB medium were pelleted by centrifugation, and RNA was extracted from the samples using the RNeasy mini kit (Qiagen) according to the manufacturer's instruction. Residual DNA was removed using the TURBO DNA‐free kit (Ambion) according to the manufacturer's instructions. The transcriptional start sites of *gvpA1* and *gvrA* were determined using a 5′/3′RACE kit (Roche). Reverse transcription PCR was carried out to determine if the left gene cluster is operonic. The synthesis of cDNA was performed with random hexamers using SuperScript II (Life Technologies). PCR was conducted using cDNA as a template with oligonucleotide pairs: RTGVL1/RTGVL2, RTGVL3/RTGVL4, RTGVL5/RTGVL6, RTGVL7/RTGVL8, RTGVL9/RTGVL10, RTGVL11/RTGVL12, RTGVL13/RTGVL14, RTGVL15/RTGVL16 and RTGVL17/RTGVL18.

The transcriptional start site of *gvpA1* was determined by 5′RACE using a 5′/3′ RACE kit (Roche). The synthesis of cDNA was carried out with an oligonucleotide RTgvpCR and cDNA was treated with terminal transferase and dATP as per the manufacturer's instruction. PCR for 5′RACE was carried out using specific oligonucleotides complementary for the *gvpA1* (gvpA1R‐HindIII) and oligo‐dT. The PCR product was cloned into pBLUEScript SK+, yielding pBluescript + gvpA1‐5′RACE. Both the original and cloned 5′RACE products were sequenced.

5′RACE analysis was also used to identify the transcriptional start site of *gvrA* and to determine if the right gene cluster was operonic. cDNA synthesis was carried out using an oligonucleotide complementary to the 3′ end of *gvrC*, the final gene in the predicted operon containing *gvrA*, *gvpH*, *gvpZ*, *gvpF2*, *gvpF3*, *gvrB* and *gvrC*. cDNA was treated with terminal transferase and dGTP to attach a poly‐G tract to the 3′ of the cDNA. PCR for 5′RACE was carried out using specific oligonucleotides complementary to *gvrA* (gvrASP1bam) and a poly‐C oligonucleotide (polyDRACE). Nested oligonucleotides were then used to further amplify from this reaction (gvrASP2bam and Anchorprimer, see Roche instructions), which resulted in amplification of a single product that was subsequently cloned into pBLUEScript SK+. Both the original and the cloned 5′RACE products were sequenced.

### Confirmation of gas vesicle formation

A total of 5 ml of overnight cultures was grown in 25 ml sealed universals on a roller wheel at 30°C and then used to subculture into a second 5 ml broth, which was grown for 24 h under the same conditions. For flotation assays, cultures were then kept statically at room temperature for 48 h before imaging by PCM, TEM or flotation in the universal tube was assessed. Cells from liquid cultures or agar plates were observed by PCM or TEM as described previously (Ramsay *et al*., 2011). Cells were imaged for PCM using an Olympus BX‐51 microscope with a 100x oil‐immersion lens, with a QICAM monochrome camera and qcapture pro‐6 software. For TEM, samples were observed in a FEI Tecnai G2 TEM. Images were captured with an AMT XR60B digital camera running deben software at the Cambridge University Advanced Imaging Centre. For colony morphology experiments, 3 µl of normalized culture were spotted onto LB agar plates, incubated at 30°C for 24 h and colony morphologies observed. Cells scraped from plates were suspended in PBS and observed by PCM.

### Pressure nephelometry

Pressure collapse experiments were performed largely using the same apparatus, and as previously described, in Holland and Walsby (2009). Cultures were grown overnight on a roller wheel at 30°C and then left to settle for 24 h at room temperature before application of incremental pressure using the apparatus shown in Fig. S5. As intracellular gas vesicles collapsed under increasing pressure, changes in culture turbidity were assessed. The collapse pressures of gas vesicles were determined by pressure nephelometry. The machine was set to zero with 4 ml of LB and 500 μl of the indicated culture was added and mixed. This mixture was carefully placed into the nephelometer, ensuring that 100% of gas vesicles remained intact, and the millivoltmeter set to 100. Pressure was applied as N_2_ gas in 0.05 MPa increments, up to 1 MPa, and the change in culture turbidity monitored after 20 s of equilibration using an analogue millivoltmeter. At each pressure increment, the proportion of gas vesicle remaining (***G***_p_) was calculated using the following formula:

$$G_{p}=\frac{G_{m}-G_{1\text{MPa}}}{G_{0\text{MPa}}-G_{1\text{MPa}}}$$

where *G_m_* is the measurement taken at the indicated pressure and *G*_1MPa_ and *G*_0MPa_ are the measurements of turbity taken at 1 MPa and 0 MPa respectively.The mean critical pressure (***p***_c_) was calculated by determining the pressure when ***G***_p_ = 0.5. When cultures were exposed to 0.35 M sucrose solutions, the mean apparent critical pressure (***p***_a_) was also determined by identifying the pressure when ***G***_p_ = 0.5. The turgor pressure (**p**_t_) was calculated by subtracting **p**_a_ from **p**_c_.

### Volume calculation of gas vesicles

Calculation of gas volume in cultures was performed as described in Walsby (1982). Briefly, cultures were grown as described for pressure nephelometry, and the relative change in volume was calculated using the apparatus shown in Fig. S5. The change in culture volume was recorded under pressure that caused the collapse of 100% of vesicles (0.8 MPa). The change in culture volume was normalized to the total cell numbers to determine the percentage of cell volume that was due to gas vesicles. Volumetric calculations were performed using a compression tube (Walsby, 1982; Walsby *et al*., 1992). Tubes, with a capillary 0.2 mm in internal diameter, were filled to capacity (∼2.5 ml of culture). The volume of gas vesicles was determined by observing the relative change in the meniscus inside the capillary (*L*) upon application of 0.8 MPa of pressure, via N_2_ gas, causing the collapse of all gas vesicles within the culture. A change in 1 mm of the meniscus within a capillary 0.2 mm in diameter is equivalent to 31.4 ηl. The mean gas vesicle volume per cell was calculated using the change in volume normalized to the number of cells in each culture, determined by colony counts.

### Measurements of gas vesicles

TEM images were analysed using imagej (Schneider *et al*., 2012). Each image was calibrated against its scale bar, and gas vesicles were measured. Mean heights and widths of gas vesicles were then calculated.

### β‐Galactosidase and β‐glucuronidase activity

S39006 Cultures were grown at 30°C with shaking at 250 r.p.m. and the optical densities (OD_600_) examined every 2 h. After 6 h of incubation, samples were taken and frozen at −80°C until required. Activity of β‐gluc, the protein product of *uidA*, was determined using the fluorogenic substrate, 4′‐Methylumbelliferyl–d‐glucuronide (Melford Laboratories), as described previously (Ramsay *et al*., 2011). Briefly, frozen samples were thawed and diluted to appropriate ratios where a linear increase in activity could be observed, and 10 µl of diluted samples was frozen and thawed again. Then, 100 µl of reaction buffer (400 g ml^−1^ lysozyme, 250 g ml^−1^ 4′‐Methylumbelliferyl–d‐glucuronide in PBS) was added, and fluorescence was immediately monitored (excitation 360 nm, emission 450 nm, cut‐off 435 nm) every 30 s for 30 min using a Gemini XPS plate reader. β‐Gluc expression was measured by the relative fluorescence units per second, produced from cleavage of 4′‐Methylumbelliferyl‐β‐d‐glucuronide, normalized to the optical density of the culture (RFU s^−1^ OD_600_^−1^). β‐Gal activity was monitored in a similar fashion but using 4′‐Methylumbelliferyl‐β‐d‐galactopyranoside (Melford Laboratories) as a fluorogenic substrate.

### Bioinformatic analyses

Sequences were accessed from NCBI, and putative gas vesicle clusters in other organisms were identified using psi‐blast (Altschul *et al*., 1997). Amino acid sequences were aligned using clustalx (Larkin *et al*., 2007) and phylogenetic trees were constructed by njplot (Perriere and Gouy, 1996). Phylogenetic distances were determined by neighbour‐joining analysis. The numbers on branching points are percentages of bootstrap values with 1000 replicates.

## Supporting information


**Fig. S1.** Phylogenetic relationships of GvpA, GvpF/L, GvpC, GvpG, GvpH, GvpK, GvpN, GvpV and GvpZ.
**Fig. S2.** The gas vesicle production locus of S39006 is composed of two operons. (A) The locus of the amplified region in the *gvp* cluster. (B) RT‐PCR results showing that the left cluster, starting from *gvpA1*, is operonic. (C) RT‐PCR result showing that there is no read through between the left and right operon. indicates the marker lane, with indicated sizes on the side. P indicates a sample including reverse transcriptase, C indicates a positive control sample containing only genomic DNA, and N indicates a negative control sample with no reverse transcriptase. (D) SmaR does not regulate *gvrA* directly. *Escherichia coli* strains carrying pRW50‐*gvrA_pro_* were grown in the presence of either pQE80 or pQE80‐SmaR with added DMSO or 1 μM BHL dissolved in DMSO, where indicated. After 8 h of growth, samples were taken and β‐gal activity assayed (represented as RFU OD_600_^−1^). The values are the average of three biological replicates ± SD.
**Fig. S3.** Complementation of GV formation in in frame mutations. Mutations in the GV cluster that failed to produce GVs were grown with the indicated plasmid, with or without 0.1 mM IPTG, and the cells were observed by PCM, flotation assays and colony morphology. (A) Analyses of Δ*gvpA1*, Δ*gvpA2* and Δ*gvpA3* mutants. (B) Analyses of Δ*gvpF1*, Δ*gvpF* and Δ*gvpF3* mutants. (C) Analyses of Δ*gvpG* and Δ*gvpK* mutants. (D) Analyses of Δ*gvpN*, Δ*gvpV*, Δ*gvrA*, Δ*gvrB* and Δ*gvrC* mutants. Scale bar indicates 1 μm.
**Fig. S4.** Measurements of GVs in different mutant strains and with application of pressure. (A) *gvpV* or *gvpN* mutant strains were imaged by TEM with or without 1.0 MPa pressure or with either *gvpV* or *gvpN* added back *in trans*. Scale bars indicate 500 nm. (B) Measurement of GVs in different strains. TEM images of GVs from the indicated strains, grown in sealed universals, were analysed using imagej and the height and widths of the indicated number (below) of vesicles measured. The values presented are the average heights and widths ± SD.
**Fig. S5.** (A) Restoration of the collapse pressure phenotype in the *gvpC* mutant, by an *in trans* copy of *gvpC*. (B–C) Apparatus used for pressure nephelometry (B) or volumetric calculations (C).
**Fig. S6.** Expression from the *gvpA1::uidA* transcriptional fusion throughout growth in S39006 WT, Δ*gvrA*, Δ*gvrB*, and Δ*gvrC* backgrounds. Dashed lines show growth and solid lines show gene expression levels via reporter enzymes. β‐Gluc activity was assayed and represented as RFU OD_600_^−1^. Values are the mean of three biological replicates ± SD.

**Table S1.** Comparison of amino acid sequences of GvpA and GvpF proteins from S39006.

**Table S2.** Bacterial strains and plasmids used in this study.

**Table S3.** Oligonucleotides used in this study.
