# Supplementary material for: Molecular genetic and physical analysis of gas vesicles in buoyant enterobacteria
Source: Environ Microbiol. 2016 Feb 15;18(4):1264–76. doi: 10.1111/1462-2920.13203 (PMC4982088; doi:10.1111/1462-2920.13203)
Supplement: Supplementary file 1 — Fig. S1. Phylogenetic relationships of GvpA, GvpF/L, GvpC, GvpG, GvpH, GvpK, GvpN, GvpV and GvpZ. Fig. S2. The gas vesicle production locus of S39006 is composed of two operons. (A) The locus of the amplified region in the gvp cluster. (B) RT‐PCR results showing that the left cluster, starting from gvpA1, is operonic. (C) RT‐PCR result showing that there is no read through between the left and right operon. indicates the marker lane, with indicated sizes on the side. P indicates a sample including reverse transcriptase, C indicates a positive control sample containing only genomic DNA, and N indicates a negative control sample with no reverse transcriptase. (D) SmaR does not regulate gvrA directly. Escherichia coli strains carrying pRW50‐gvrApro were grown in the presence of either pQE80 or pQE80‐SmaR with added DMSO or 1 μM BHL dissolved in DMSO, where indicated. After 8 h of growth, samples were taken and β‐gal activity assayed (represented as RFU OD600 −1). The values are the average of three biological replicates ± SD. Fig. S3. Complementation of GV formation in in frame mutations. Mutations in the GV cluster that failed to produce GVs were grown with the indicated plasmid, with or without 0.1 mM IPTG, and the cells were observed by PCM, flotation assays and colony morphology. (A) Analyses of ΔgvpA1, ΔgvpA2 and ΔgvpA3 mutants. (B) Analyses of ΔgvpF1, ΔgvpF and ΔgvpF3 mutants. (C) Analyses of ΔgvpG and ΔgvpK mutants. (D) Analyses of ΔgvpN, ΔgvpV, ΔgvrA, ΔgvrB and ΔgvrC mutants. Scale bar indicates 1 μm. Fig. S4. Measurements of GVs in different mutant strains and with application of pressure. (A) gvpV or gvpN mutant strains were imaged by TEM with or without 1.0 MPa pressure or with either gvpV or gvpN added back in trans. Scale bars indicate 500 nm. (B) Measurement of GVs in different strains. TEM images of GVs from the indicated strains, grown in sealed universals, were analysed using imagej and the height and widths of the indicated number (below) of vesicles [file EMI-18-1264-s001.pdf]

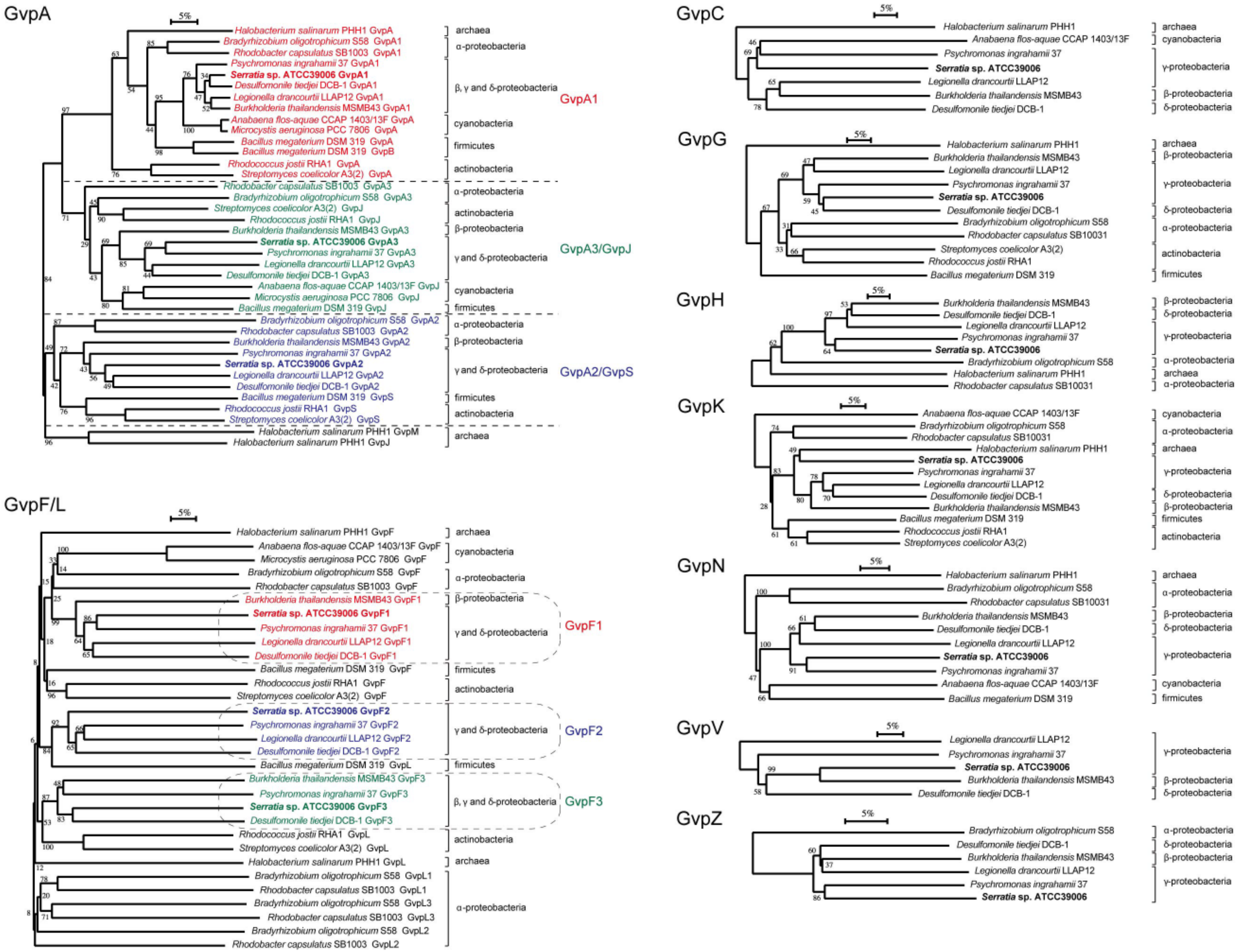
GvpA
5%
Halobacterium salinarum PHH1 GvpA
Bradyrhizobium oligotrophicum S58 GvpA1
Rhodobacter capsulatus SB1003 GvpA1
Psychromonas ingrahamii 37 GvpA1
Serratia sp. ATCC39006 GvpA1
Desulfomonile tiedjei DCB-1 GvpA1
Legionella drancourtii LLAP12 GvpA1
Burkholderia thailandensis MSMB43 GvpA1
Anabaena flos-aquae CCAP 1403/13F GvpA
Microcystis aeruginosa PCC 7806 GvpA
Bacillus megaterium DSM 319 GvpA
Bacillus megaterium DSM 319 GvpB
Rhodococcus jostii RHA1 GvpA
Streptomyces coelicolor A3(2) GvpA
Rhodobacter capsulatus SB1003 GvpA3
Bradyrhizobium oligotrophicum S58 GvpA3
Streptomyces coelicolor A3(2) GvpJ
Rhodococcus jostii RHA1 GvpJ
Burkholderia thailandensis MSMB43 GvpA3
Serratia sp. ATCC39006 GvpA3
Psychromonas ingrahamii 37 GvpA3
Legionella drancourtii LLAP12 GvpA3
Desulfomonile tiedjei DCB-1 GvpA3
Anabaena flos-aquae CCAP 1403/13F GvpJ
Microcystis aeruginosa PCC 7806 GvpJ
Bacillus megaterium DSM 319 GvpJ
Bradyrhizobium oligotrophicum S58 GvpA2
Rhodobacter capsulatus SB1003 GvpA2
Burkholderia thailandensis MSMB43 GvpA2
Psychromonas ingrahamii 37 GvpA2
Serratia sp. ATCC39006 GvpA2
Legionella drancourtii LLAP12 GvpA2
Desulfomonile tiedjei DCB-1 GvpA2
Bacillus megaterium DSM 319 GvpS
Rhodococcus jostii RHA1 GvpS
Streptomyces coelicolor A3(2) GvpS
Halobacterium salinarum PHH1 GvpM
Halobacterium salinarum PHH1 GvpJ
archaea
α-proteobacteria
β, γ and δ-proteobacteria
cyanobacteria
firmicutes
actinobacteria
α-proteobacteria
actinobacteria
β-proteobacteria
γ and δ-proteobacteria
cyanobacteria
firmicutes
α-proteobacteria
β-proteobacteria
γ and δ-proteobacteria
firmicutes
actinobacteria
archaea
GvpA1
GvpA3/GvpJ
GvpA2/GvpS
GvpF/L
5%
Halobacterium salinarum PHH1 GvpF
Anabaena flos-aquae CCAP 1403/13F GvpF
Microcystis aeruginosa PCC 7806 GvpF
Bradyrhizobium oligotrophicum S58 GvpF
Rhodobacter capsulatus SB1003 GvpF
Burkholderia thailandensis MSMB43 GvpF1
Serratia sp. ATCC39006 GvpF1
Psychromonas ingrahamii 37 GvpF1
Legionella drancourtii LLAP12 GvpF1
Desulfomonile tiedjei DCB-1 GvpF1
Bacillus megaterium DSM 319 GvpF
Rhodococcus jostii RHA1 GvpF
Streptomyces coelicolor A3(2) GvpF
Serratia sp. ATCC39006 GvpF2
Psychromonas ingrahamii 37 GvpF2
Legionella drancourtii LLAP12 GvpF2
Desulfomonile tiedjei DCB-1 GvpF2
Bacillus megaterium DSM 319 GvpL
Burkholderia thailandensis MSMB43 GvpF3
Psychromonas ingrahamii 37 GvpF3
Serratia sp. ATCC39006 GvpF3
Desulfomonile tiedjei DCB-1 GvpF3
Rhodococcus jostii RHA1 GvpL
Streptomyces coelicolor A3(2) GvpL
Halobacterium salinarum PHH1 GvpL
Bradyrhizobium oligotrophicum S58 GvpL1
Rhodobacter capsulatus SB1003 GvpL1
Bradyrhizobium oligotrophicum S58 GvpL3
Rhodobacter capsulatus SB1003 GvpL3
Bradyrhizobium oligotrophicum S58 GvpL2
Rhodobacter capsulatus SB1003 GvpL2
archaea
cyanobacteria
α-proteobacteria
β-proteobacteria
γ and δ-proteobacteria
firmicutes
actinobacteria
γ and δ-proteobacteria
firmicutes
β, γ and δ-proteobacteria
actinobacteria
archaea
α-proteobacteria
GvpF1
GvpF2
GvpF3
GvpC
5%
Halobacterium salinarum PHH1
Anabaena flos-aquae CCAP 1403/13F
Psychromonas ingrahamii 37
Serratia sp. ATCC39006
Legionella drancourtii LLAP12
Burkholderia thailandensis MSMB43
Desulfomonile tiedjei DCB-1
archaea
cyanobacteria
γ-proteobacteria
β-proteobacteria
δ-proteobacteria
GvpG
5%
Halobacterium salinarum PHH1
Burkholderia thailandensis MSMB43
Legionella drancourtii LLAP12
Psychromonas ingrahamii 37
Serratia sp. ATCC39006
Desulfomonile tiedjei DCB-1
Bradyrhizobium oligotrophicum S58
Rhodobacter capsulatus SB10031
Streptomyces coelicolor A3(2)
Rhodococcus jostii RHA1
Bacillus megaterium DSM 319
archaea
β-proteobacteria
γ-proteobacteria
δ-proteobacteria
α-proteobacteria
actinobacteria
firmicutes
GvpH
5%
Burkholderia thailandensis MSMB43
Desulfomonile tiedjei DCB-1
Legionella drancourtii LLAP12
Psychromonas ingrahamii 37
Serratia sp. ATCC39006
Bradyrhizobium oligotrophicum S58
Halobacterium salinarum PHH1
Rhodobacter capsulatus SB10031
β-proteobacteria
δ-proteobacteria
γ-proteobacteria
α-proteobacteria
archaea
α-proteobacteria
GvpK
5%
Anabaena flos-aquae CCAP 1403/13F
Bradyrhizobium oligotrophicum S58
Rhodobacter capsulatus SB10031
Halobacterium salinarum PHH1
Serratia sp. ATCC39006
Psychromonas ingrahamii 37
Legionella drancourtii LLAP12
Desulfomonile tiedjei DCB-1
Burkholderia thailandensis MSMB43
Bacillus megaterium DSM 319
Rhodococcus jostii RHA1
Streptomyces coelicolor A3(2)
cyanobacteria
α-proteobacteria
archaea
γ-proteobacteria
δ-proteobacteria
β-proteobacteria
firmicutes
actinobacteria
GvpN
5%
Halobacterium salinarum PHH1
Bradyrhizobium oligotrophicum S58
Rhodobacter capsulatus SB10031
Burkholderia thailandensis MSMB43
Desulfomonile tiedjei DCB-1
Legionella drancourtii LLAP12
Serratia sp. ATCC39006
Psychromonas ingrahamii 37
Anabaena flos-aquae CCAP 1403/13F
Bacillus megaterium DSM 319
archaea
α-proteobacteria
β-proteobacteria
δ-proteobacteria
γ-proteobacteria
cyanobacteria
firmicutes
GvpV
5%
Legionella drancourtii LLAP12
Psychromonas ingrahamii 37
Serratia sp. ATCC39006
Burkholderia thailandensis MSMB43
Desulfomonile tiedjei DCB-1
γ-proteobacteria
β-proteobacteria
δ-proteobacteria
GvpZ
5%
Bradyrhizobium oligotrophicum S58
Desulfomonile tiedjei DCB-1
Burkholderia thailandensis MSMB43
Legionella drancourtii LLAP12
Psychromonas ingrahamii 37
Serratia sp. ATCC39006
α-proteobacteria
δ-proteobacteria
β-proteobacteria
γ-proteobacteria

Figure S1

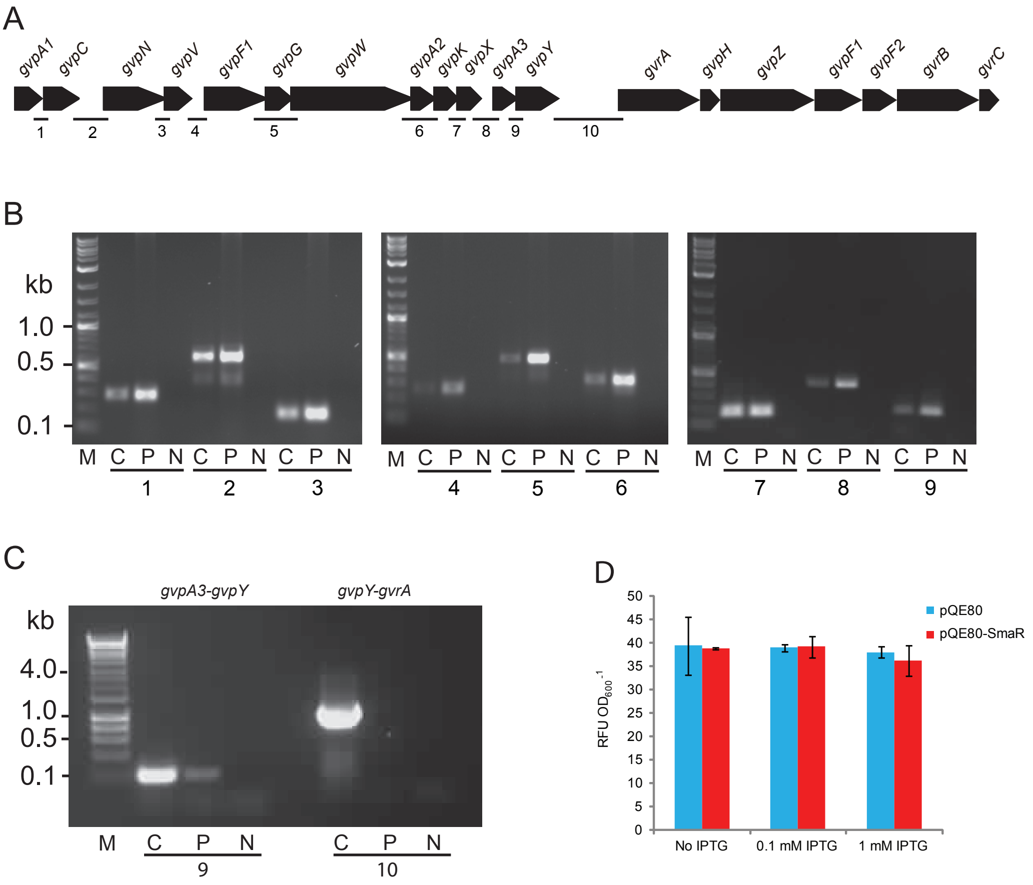
A
gvpA1
gvpC
gvpN
gvpV
gvpF1
gvpG
gvpW
gvpA2
gvpK
gvpX
gvpA3
gvpY
gvrA
gvpH
gvpZ
gvpF1
gvpF2
gvrB
gvrC
1 2 3 4 5 6 7 8 9 10
B
kb
1.0
0.5
0.1
M C P N C P N C P N
1 2 3
M C P N C P N C P N
4 5 6
M C P N C P N C P N
7 8 9
C
gvpA3-gvpY
gvpY-gvrA
kb
4.0
1.0
0.5
0.1
M C P N C P N
9 10
D
pQE80
pQE80-SmaR
RFU OD600^-1
50 45 40 35 30 25 20 15 10 5 0
No IPTG
0.1 mM IPTG
1 mM IPTG

Figure S2

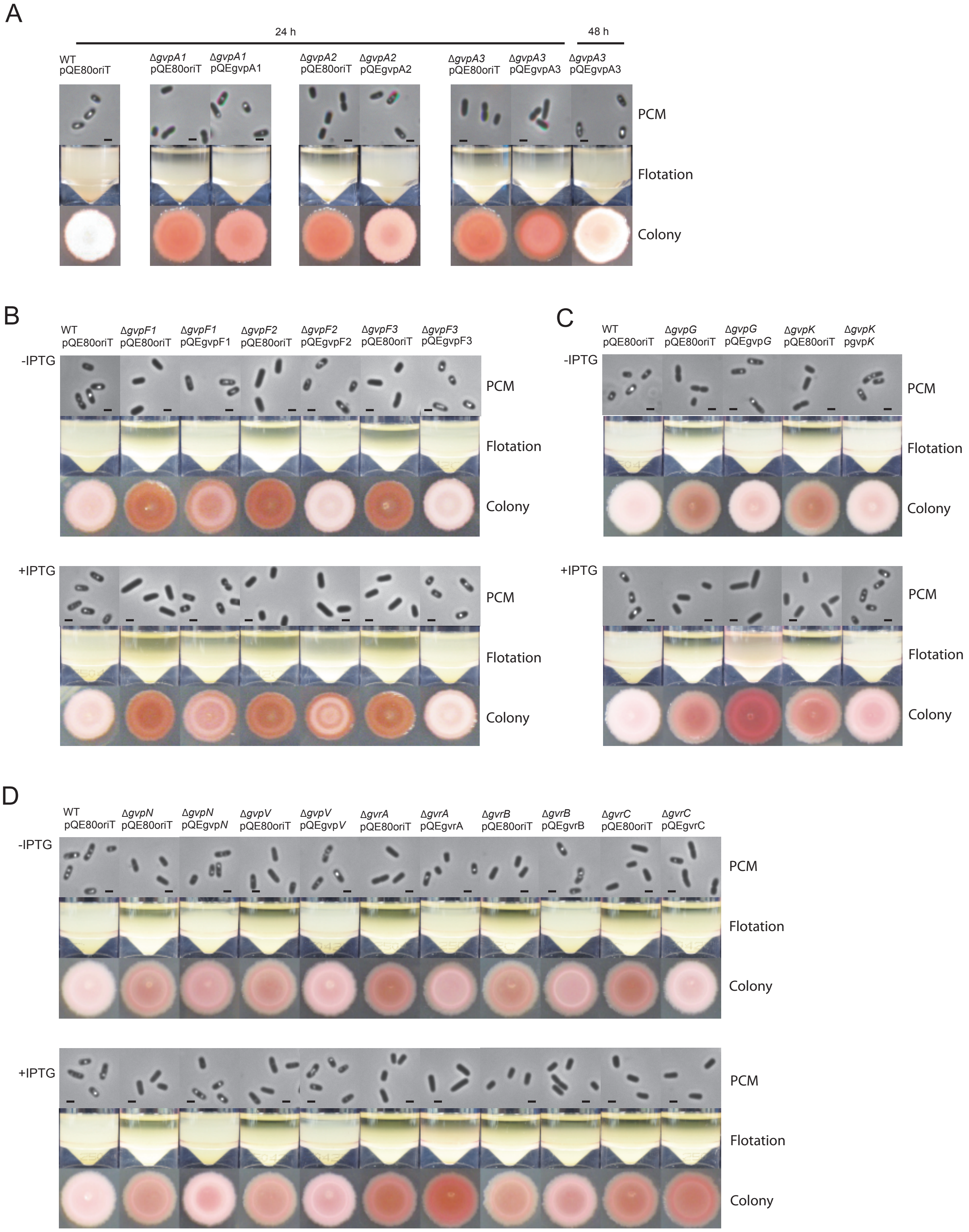
A
24 h
48 h
WT pQE80oriT
ΔgvpA1 pQE80oriT
ΔgvpA1 pQEgvpA1
ΔgvpA2 pQE80oriT
ΔgvpA2 pQEgvpA2
ΔgvpA3 pQE80oriT
ΔgvpA3 pQEgvpA3
ΔgvpA3 pQEgvpA3
PCM
Flotation
Colony
B
WT pQE80oriT
ΔgvpF1 pQE80oriT
ΔgvpF1 pQEgvpF1
ΔgvpF2 pQE80oriT
ΔgvpF2 pQEgvpF2
ΔgvpF3 pQE80oriT
ΔgvpF3 pQEgvpF3
-IPTG
+IPTG
PCM
Flotation
Colony
C
WT pQE80oriT
ΔgvpG pQE80oriT
ΔgvpG pQEgvpG
ΔgvpK pQE80oriT
ΔgvpK pgvpK
-IPTG
+IPTG
PCM
Flotation
Colony
D
WT pQE80oriT
ΔgvpN pQE80oriT
ΔgvpN pQEgvpN
ΔgvpV pQE80oriT
ΔgvpV pQEgvpV
ΔgvrA pQE80oriT
ΔgvrA pQEgvrA
ΔgvrB pQE80oriT
ΔgvrB pQEgvrB
ΔgvrC pQE80oriT
ΔgvrC pQEgvrC
-IPTG
+IPTG
PCM
Flotation
Colony

Figure S3

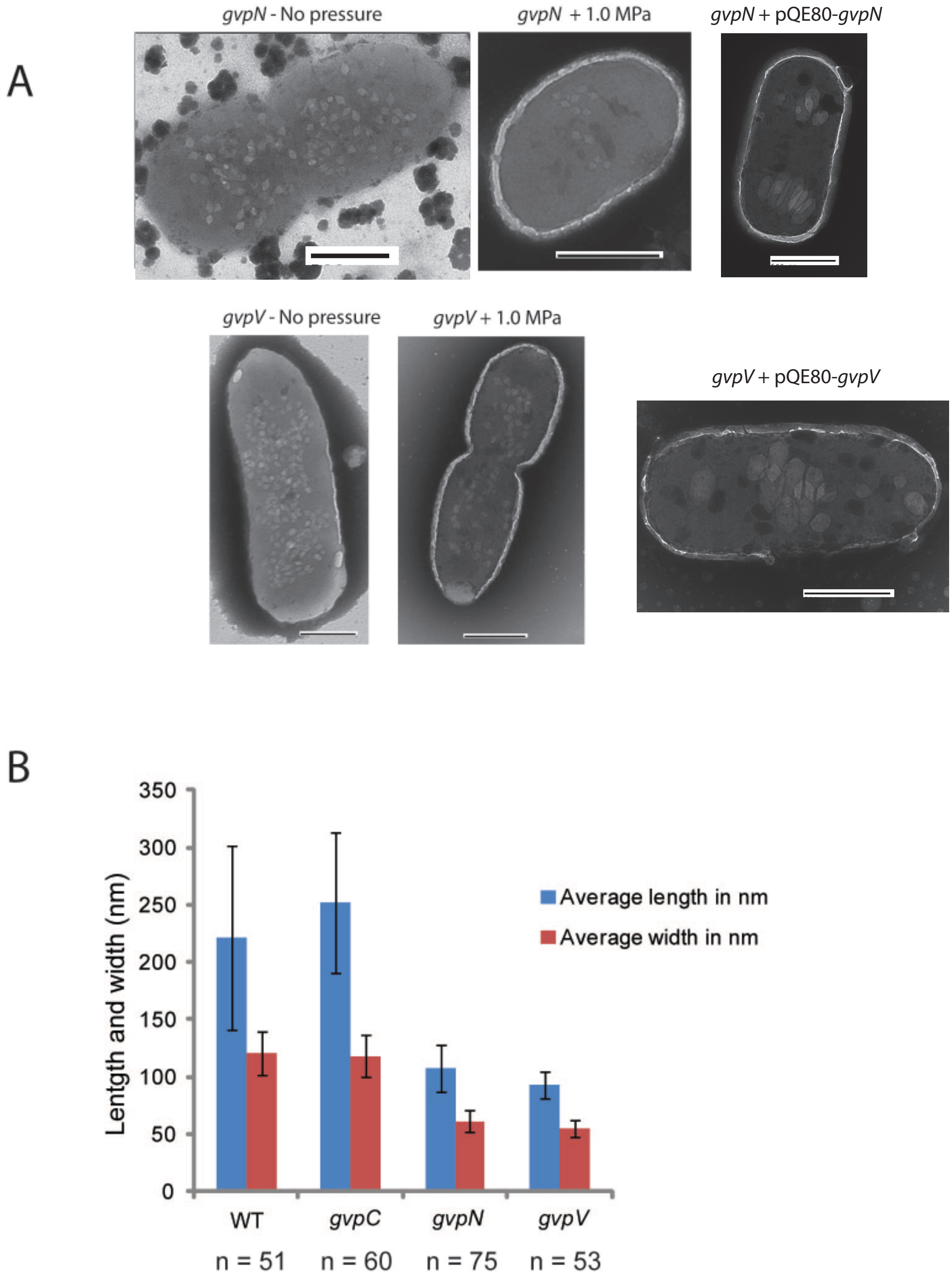

A
gvpN - No pressure
gvpN + 1.0 MPa
gvpN + pQE80-gvpN
gvpV - No pressure
gvpV + 1.0 MPa
gvpV + pQE80-gvpV
B
Lentgth and width (nm)
350
300
250
200
150
100
50
0
Average length in nm
Average width in nm
WT
gvpC
gvpN
gvpV
n = 51
n = 60
n = 75
n = 53

Figure S4

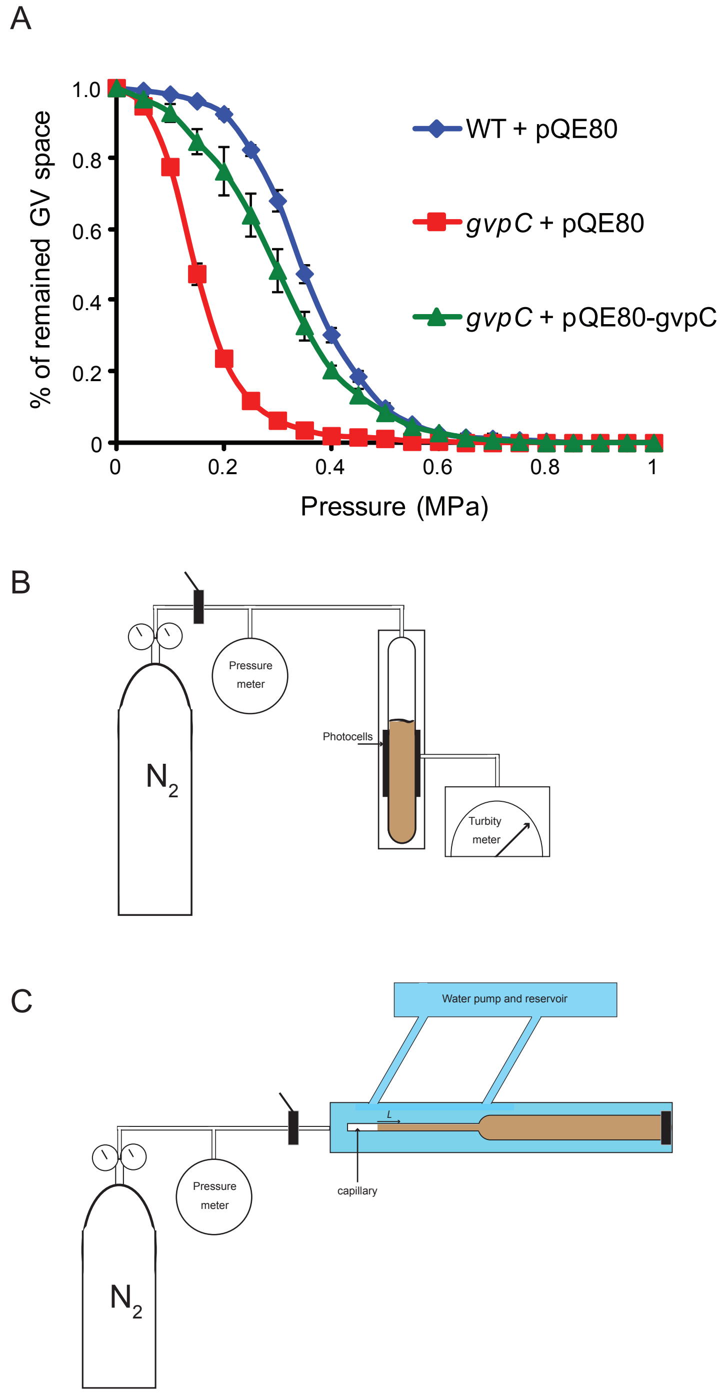
A
1.0
0.8
0.6
0.4
0.2
0
% of remained GV space
0
0.2
0.4
0.6
0.8
1
Pressure (MPa)
WT + pQE80
gvpC + pQE80
gvpC + pQE80-gvpC
B
Pressure meter
N2
Photocells
Turbity meter
C
Water pump and reservoir
L
capillary
Pressure meter
N2

Figure S5

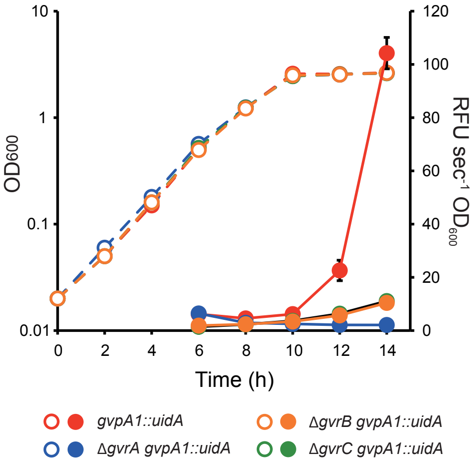

10
1
0.1
0.01
OD600
120
100
80
60
40
20
0
RFU sec-1 OD600
0
2
4
6
8
10
12
14
Time (h)
gvpA1::uidA
ΔgvrA gvpA1::uidA
ΔgvrB gvpA1::uidA
ΔgvrC gvpA1::uidA

Figure S6
