# Supplementary material for: Molecular genetic and physical analysis of gas vesicles in buoyant enterobacteria
Source: Environ Microbiol. 2016 Feb 15;18(4):1264–76. doi: 10.1111/1462-2920.13203 (PMC4982088; doi:10.1111/1462-2920.13203)
Supplement: Supplementary file 2 — Table S1. Comparison of amino acid sequences of GvpA and GvpF proteins from S39006. [file EMI-18-1264-s002.pdf]

Table S1 Comparison of Amino Acid Sequences of GvpA and GvpF proteins from S39006

A)Comparison of GvpA amino acid sequences from S39006

	GvpA2	GvpA3
GvpA1	(35% , 64%)	(39%, 71%)
GvpA2		(34%, 60%)

B) Comparison of GvpF amino acid sequences from S39006

	GvpF2	GvpF3
GvpF1	(23%, 43%)	(22%, 54%)
GvpF2		(21%, 37%)

Numbers indicate (% identity, % similarity)
