# Supplementary material for: Molecular genetic and physical analysis of gas vesicles in buoyant enterobacteria
Source: Environ Microbiol. 2016 Feb 15;18(4):1264–76. doi: 10.1111/1462-2920.13203 (PMC4982088; doi:10.1111/1462-2920.13203)
Supplement: Supplementary file 3 — Table S2. Bacterial strains and plasmids used in this study. [file EMI-18-1264-s003.pdf]

Table S2 Bacterial strains and plasmids used in this study

Strains/plasmids	Genotype or discription	Reference
Serratia sp.		
ATCC39006	From ATCC	Parker WL 1982
LacA (WT)	Laboratory strain, referred to as wild type (WT) in text, Lac-derivative of ATCC S39006	
Δ*gvpA1*	In-frame *gvpA1* mutant derivative of WT, made using plasmid pKNG101-ΔgvpA1	This study
Δ*gvpA2*	In-frame *gvpA2* mutant derivative of WT, made using plasmid pKNG101-ΔgvpA2	This study
Δ*gvpA3*	In-frame *gvpA3* mutant derivative of WT, made using plasmid pKNG101-ΔgvpA3	This study
Δ*gvpC*	In-frame *gvpC* mutant derivative of WT, made using plasmid pKNG101-ΔgvpC	This study
Δ*gvpF1*	In-frame *gvpF1* mutant derivative of WT, made using plasmid pKNG101-ΔgvpF1	This study
Δ*gvpF2*	In-frame *gvpF2* mutant derivative of WT, made using plasmid pKNG101-ΔgvpF2	This study
Δ*gvpF3*	In-frame *gvpF3* mutant derivative of WT, made using plasmid pKNG101-ΔgvpF3	This study
Δ*gvpG*	In-frame *gvpG* mutant derivative of WT, made using plasmid pKNG101-ΔgvpG	This study
Δ*gvpH*	In-frame *gvpH* mutant derivative of WT, made using plasmid pKNG101-ΔgvpH	This study
Δ*gvpK*	In-frame *gvpK* mutant derivative of WT, made using plasmid pKNG101-ΔgvpK	This study
Δ*gvpN*	In-frame *gvpN* mutant derivative of WT, made using plasmid pKNG101-ΔgvpN	This study
Δ*gvpV*	In-frame *gvpV* mutant derivative of WT, made using plasmid pKNG101-ΔgvpV	This study
Δ*gvpW*	In-frame *gvpW* mutant derivative of WT, made using plasmid pKNG101-ΔgvpW	This study
Δ*gvpX*	In-frame *gvpX* mutant derivative of WT, made using plasmid pKNG101-ΔgvpX	This study
Δ*gvpY*	In-frame *gvpY* mutant derivative of WT, made using plasmid pKNG101-ΔgvpY	This study
Δ*gvpZ*	In-frame *gvpZ* mutant derivative of WT, made using plasmid pKNG101-ΔgvpZ	This study
Δ*gvrA*	In-frame *gvrA* mutant derivative of WT, made using plasmid pKNG101-ΔgvrA	This study
Δ*gvrB*	In-frame *gvrB* mutant derivative of WT, made using plasmid pKNG101-ΔgvrB	This study
Δ*gvrC*	In-frame *gvrC* mutant derivative of WT, made using plasmid pKNG101-ΔgvrC	This study
GPA1	*gvpA1* ::Tn-DS1028-*uidA* derivative of WT, Cm^R	Ramsay JP 2011
Δ*gvrA gvpA1::uidA*	In-frame *gvrA* mutant derivative of GPA1	This study
Δ*gvrB gvpA1::uidA*	In-frame *gvrB* mutant derivative of GPA1	This study
Δ*gvrC gvpA1::uidA*	In-frame *gvrC* mutant derivative of GPA1	This study
Esherichia coli		
DH5α	*supE44* , Δ*lac* U169, Φ80*lacZ* ΔM15, *hsdR17* , *recA1* , *endA1* , *gyrA96* , *thi-1, relA1* , *luxS*	
β2163	(F−) RP4-2-Tc::Mu *dapA* ::(*erm* -*pir*)	
Plasmids		
pQE80oriT	Expression vector pQE80L containing the RK2 origin, Ap^R	Ramsay JP 2011
pKNG101	Marker exchange suicide vector, *sacBR* , mobRK2, oriR6K, Sm^R	Kaniga 1991
pKNG101-ΔgvpA1	pKNG101 marker exchange construct containing Δ*gvpA1*	This study
pKNG101-ΔgvpA2	pKNG101 marker exchange construct containing Δ*gvpA2*	This study
pKNG101-ΔgvpA3	pKNG101 marker exchange construct containing Δ*gvpA3*	This study
pKNG101-ΔgvpC	pKNG101 marker exchange construct containing Δ*gvpC*	This study
pKNG101-ΔgvpF1	pKNG101 marker exchange construct containing Δ*gvpF1*	This study
pKNG101-ΔgvpF2	pKNG101 marker exchange construct containing Δ*gvpF2*	This study
pKNG101-ΔgvpF3	pKNG101 marker exchange construct containing Δ*gvpF3*	This study
pKNG101-ΔgvpG	pKNG101 marker exchange construct containing Δ*gvpG*	This study
pKNG101-ΔgvpH	pKNG101 marker exchange construct containing Δ*gvpH*	This study
pKNG101-ΔgvpK	pKNG101 marker exchange construct containing Δ*gvpK*	This study
pKNG101-ΔgvpN	pKNG101 marker exchange construct containing Δ*gvpN*	This study
pKNG101-ΔgvpV	pKNG101 marker exchange construct containing Δ*gvpV*	This study
pKNG101-ΔgvpW	pKNG101 marker exchange construct containing Δ*gvpW*	This study
pKNG101-ΔgvpX	pKNG101 marker exchange construct containing Δ*gvpX*	This study
pKNG101-ΔgvpY	pKNG101 marker exchange construct containing Δ*gvpY*	This study
pKNG101-ΔgvpZ	pKNG101 marker exchange construct containing Δ*gvpZ*	This study
pKNG101-ΔgvrA	pKNG101 marker exchange construct containing Δ*gvrA*	This study
pKNG101-ΔgvrB	pKNG101 marker exchange construct containing Δ*gvrB*	This study
pKNG101-ΔgvrC	pKNG101 marker exchange construct containing Δ*gvrC*	This study
pQE80oriT-gvpA1	pQE80oriT containing SD sequence and ORF of *gvpA1* as a EcoRI-HindII fragment	This study
pQE80oriT-gvpA2	pQE80oriT containing SD sequence and ORF of *gvpA2* as a EcoRI-HindII fragment	This study
pQE80oriT-gvpA3	pQE80oriT containing SD sequence and ORF of *gvpA3* as a EcoRI-HindII fragment	This study
pQE80oriT-gvpC	pQE80oriT containing SD sequence and ORF of *gvpC* as a EcoRI-HindII fragment	This study
pQE80oriT-gvpF1	pQE80oriT containing SD sequence and ORF of *gvpF1* as a EcoRI-HindII fragment	This study
pQE80oriT-gvpF2	pQE80oriT containing SD sequence and ORF of *gvpF2* as a EcoRI-SalI fragment	This study
pQE80oriT-gvpF3	pQE80oriT containing SD sequence and ORF of *gvpF3* as a EcoRI-HindII fragment	This study
pQE80oriT-gvpG	pQE80oriT containing SD sequence and ORF of *gvpG* as a EcoRI-HindII fragment	This study
pQE80oriT-gvpK	pQE80oriT containing SD sequence and ORF of *gvpK* as a EcoRI-HindII fragment	This study
pQE80oriT-gvpN	pQE80oriT containing SD sequence and ORF of *gvpN* as a EcoRI-HindII fragment	This study
pQE80oriT-gvpV	pQE80oriT containing SD sequence and ORF of *gvpV* as a EcoRI-HindII fragment	This study
pQE80oriT-gvrA	pQE80oriT containing SD sequence and ORF of *gvrA* as a EcoRI-HindII fragment	This study
pQE80oriT-gvrB	pQE80oriT containing SD sequence and ORF of *gvrB* as a BamHI-HindII fragment	This study
pQE80oriT-gvrC	pQE80oriT containing SD sequence and ORF of *gvrC* as a EcoRI-HindII fragment	This study
pQE80-SmaR	pQE80 plasmid containing SmaR, Ap^R	Slater et al. 2003
pBLUEScript SK+	Cloning vector, ColE1 replicon, Ap^R	Stratagene
pBluescript +gvpA1 5'RAC	pBLUEScript SK+ containing the product of 5'RACE beginning at the transcription start site of *gvpA1*	This study
pBluescript +gvrA 5'RACE	pBLUEScript SK+ containing the product of 5'RACE beginning at the transcription start site of *gvrA*	This study
pRW50	promoterless *lacZ* reporter, Tet^R	Lodge et al. 1992
pRW50-*gvpA1* $_{pro}$	pRW50 derivative containing *gvpA1* promoter as an EcoRI/HindIII fragment	This study
pRW50-*gvrA* $_{pro}$	pRW50 derivative containing *gvrA* promoter as an EcoRI/HindIII fragment	This study
