# Supplementary material for: Molecular genetic and physical analysis of gas vesicles in buoyant enterobacteria
Source: Environ Microbiol. 2016 Feb 15;18(4):1264–76. doi: 10.1111/1462-2920.13203 (PMC4982088; doi:10.1111/1462-2920.13203)
Supplement: Supplementary file 4 — Table S3. Oligonucleotides used in this study. [file EMI-18-1264-s004.pdf]

Table S3 Oligonucleotides used in this study

Oligonucleotide	Sequences (5'-3') (underlined is restriction enzyme site)
gvpA1_5F_BamHI	CGGGATCCATCCCTGTAATCTTGCAC
gvpA1_5R_XhoI	ATTACGCCTCGAGAGCCATGATGGCGTCTCC
gvpA1_3F_XhoI	CATGGCTCTCGAGGCGTAATTAAGTAAGCAG
gvpA1_3R_SpeI	CTACACTAGTGCTGCCAAGTGCCAACG
gvpA2_5F_BamHI	CGGGATCCGCTGATTTTGACAAAATCCG
gvpA2_5R_XhoI	GTTACTCCTCGAGTGGCATAATGTGTACGCTC
gvpA2_3F_XhoI	TATGCCACTCGAGGAGTAACACTATGACAACC
gvpA2_3R_SpeI	CTACACTAGTCTGGCGATATCGCTTGCG
gvpA3_5F_SalI	ACGCGTCGACGGCCGATTGCTGTAAGG
gvpA3_5R_XhoI	AATCCTTACTCGAGGCTCATGGTGCAAATCC
gvpA3_3F_XhoI	ATGAGCCTCGAGTAAGGATTATCTTTTTTATGCG
gvpA3_3R_SpeI	CTACACTAGTATTGCTGCTATGCCGATAC
gvpF1_5F_BamHI	CGGGATCCTAATCAGAGAGTTGCGTGG
gvpF1_5R_XhoI	GTCAGACTCGAGAATACTCATCATGCACTCC
gvpF1_3F_XhoI	GAGTATTCTCGAGTCTGACTGAGGCTAAGCC
gvpF1_3R_SpeI	CTACACTAGTGCCAATTCATCCATGCC
gvpF2_5F_BamHI	CGGGATCCCGGTTTGGATGACGTACG
gvpF2_5R_XhoI	GTAGGCCTCGAGTTCTGTGTTCATCGTCTAGC
gvpF2_3F_XhoI	CACAGAACTCGAGGCCTACAGTTTTTGCCAAC
gvpF2_3R_SpeI	CTACACTAGTCATGCAGTTTCTCATCC
gvpF3_5F_BamHI	CGGGATCCTTTGCTGAAGTGATTGCCG
gvpF3_5R_XhoI	GGGAGGCTCGAGGCCGTACAGTAGTAAGCTC
gvpF3_3F_XhoI	GTACGGCCTCGAGCCTCCCTATAACTTCGCC
gvpF3_3R_SpeI	CTACACTAGTCAGTTCCAATTTCCGACG
gvpC_5F_BamHI	CGGGATCCGTAAGGGCGACATCATGACC
gvpC_5R_XhoI	GCTCGTCTCGAGACCGTCTGTTAAGCATCC
gvpC_3F_XhoI	GACGGTCTCGAGACGAGCCTCGCAGTCTAGC
gvpC_3R_SpeI	CTACACTAGTCTTGCAACATGGTAACGG
gvpG_5F_BamHI	CGGGATCCATGCGCTCTGTGTTTCCG
gvpG_5R_XhoI	GTCGTCCTCGAGGATGTCATCGATCAGCAGC
gvpG_3F_XhoI	GACATCCTCGAGGACGACGACGAGGATGAGC
gvpG_3R_SpeI	CTACACTAGTGGAAATAGTCGATTGACC
gvpK_5F_SalI	ACGCGTCGACGGATTAGATGAAATCATGG
gvpK_5R_XhoI	GCCTAGCTCGAGATGGTGCGATAACTGTAAGG
gvpK_3F_XhoI	CACCATCTCGAGCTAGGCCGATTGCTGTAAGG
gvpK_3R_SpeI	CTACACTAGTTGATGACAACCCCTTTGTCC
gvpN_5F_BamHI	CGGGATCCACAGAGTGAAGAACGTGCC
gvpN_5R_XhoI	GTAGTTCCTCGAGCTGCGAAACGGTATTTTG
gvpN_3F_XhoI	TCGCAGCTCGAGGAACTACAGATACTGCATGC
gvpN_3R_SpeI	CTACACTAGTATACGTCCAAAATCAGCG
gvpH_5F_BamHI	CGGGATCCCGCGACTAAACCCATTCC
gvpH_5R_XhoI	CCGTTCCTCGAGCTGGTTTCCATCCAAACC
gvpH_3F_XhoI	AACCAGCTCGAGGAACGGTCTTGCCGTAATGG
gvpH_3R_SpeI	CTACACTAGTGCTGATGGTAAAAAATTGCG
gvpX_5F_SalI	ACGCGTCGACACTGTGCCAGGCAATCACG
gvpX_5R_XhoI	GGATGTCTCGAGCCTCTGCCGAAAACAGAC
gvpX_3F_XhoI	CAGAGGCTCGAGACATCCATATAAGTTTCAGG
gvpX_3R_SpeI	CTACACTAGTCACGCGTTTGAGTTTCGG
gvpV_5F_BamHI	CGGGATCCACCATGTTGCAAGGTAACG
gvpV_5R_XhoI	TCGCAACTCGAGAGGACGAGTACTTATAGCC
gvpV_3F_XhoI	CGTCCTCTCGAGTTGCGAATCAAGTATTGAGG
gvpV_3R_SpeI	CTACACTAGTGGTTTTCAATAATCTCTTCGC
gvpW_5F_BamHI	CGGGATCCCCAATGCCAATAATCACCG
gvpW_5R_XhoI	ACTCTCCTCGAGCTTGGGATAAATAGCAGG
gvpW_3F_XhoI	CCCAAGCTCGAGGAGAGTTCCATTTCGCTGC
gvpW_3R_SpeI	CTACACTAGTTTGCCTGGCACAGTGTGC
gvpY_5F_SalI	ACGCGTCGACCAACGTTATGAACGAGATGG
gvpY_5R_XhoI	CGGTTGCTCGAGAATGGGAAAATGAATCAGC
gvpY_3F_XhoI	CCCATTCTCGAGCAACCGTTAGGTAAAGGGG
gvpY_3R_SpeI	CTACACTAGTATCATCGCCTAGACGGTGC
gvpZ_5F_BamHI	CGGGATCCTCCGACAAAAGAGTAGGG
gvpZ_5R_XhoI	ACGCACCTCGAGTTCTTCAGACATGACTGCC
gvpZ_3F_XhoI	GAAGAACTCGAGGTGCGTTGGGAAGATGTCGG
gvpZ_3R_SpeI	CTACACTAGTCATCGCCAAACATTTCGGC
gvrA_5F_BamHI	CGGGATCCGTTTGTAAAAGTATCGGC
gvrA_5R_XhoI	GATCTTCTCGAGGTCACAAATATCTTTGTCG
gvrA_3F_XhoI	TGTGACCTCGAGAAGATCAATGCAGCCGAGG
gvrA_3R_SpeI	CTACACTAGTGAAGCGGGTTCAATCTGC
gvrB_5F_BamHI	CGGGATCCTGCTGACATTGTTGAACGC
gvrB_5R_XhoI	CAGACACTCGAGTGACATGGTAGCCTGTCG
gvr**B**_3F_XhoI	CATGTCACTCGAGTGTCTGGGTGAAGGTTCC
gvr**B**_3R_SpeI	CTACACTAGTCTGGTTCTATGATCTGTGG
gvrC_5F_BamHI	CGGGATCCTGAAGGATGACTATGGGC
gvrC_5R_XhoI	GTGTCTGCTCGAGAACTATCAGAATGGGTGC
gvrC_3F_XhoI	GATAGTTCTCGAGCAGACACTATCACAGCCG
gvrC_3R_SpeI	CTACACTAGTAGCGAACTGGAACGTACG
RTGVL1	ACCGTATTCTGGACAAAGG
RTGVL2	GCCATACCGTCTGTTAAGC
RTGVL3	AACGAGCCTCGCAGTCTAG
RTGVL4	ACGGTATACTGCGAAACGG
RTGVL5	ACCAATAGGTTCAGACACG
RTGVL6	ATTCCAGCGCACCTATCTG
RTGVL7	GTCTTCTGGTTTGCGAATC
RTGVL8	TTGGCACGATGATTTCTGG

Table S3 Oligonucleotides used in this study

RTGVL9	CTGGTACTCTCTCTGACTG
RTGVL10	GACTCCAGTAAGAACTTGG
RTGVL11	GCTAAAGACATTGGGTTCG
RTGVL12	TGTATTGCTGGCGATGTCG
RTGVL13	TTGACACATTTGTGCGACG
RTGVL14	TTAACCATGGTACACACCC
RTGVL15	ATATCCTTGAACGCGTTGG
RTGVL16	GGTCGAATCGGTTGAATGG
RTGVL17	GAGTTGGAAGAACGTCTGC
RTGVL18	AGATACGCACGCGTTTGAG
RTgvp R	AACGCCTCTGACATTATCGG
gvpA1F-EcoRI	GGAATTCTAGGAGACGCCATCATGGC
gvpA1R-HindIII	CCCAAGCTTAATTACGCTGGAGTCGCTGC
gvpA2F-EcoRI	GGAATTCTGTGAGCGTACACATTATGC
gvpA2R-HindIII	CCCAAGCTTACTCCCTACCGGGTAAACGG
gvpA3F-EcoRI	GGAATTCTATAAGGATTTGCACCATGAGC
gvpA3R-HindIII	CCCAAGCTTCCTTATAAATGAGTTTCTTC
gvpCF-EcoRI	GGAATTCATAAGGAGAATCCTATGGG
gvpCR-HindIII	CCCAAGCTTGGCTAGACTGCGAGGCTCG
gvpGF-EcoRI	GGAATTCTGAGGCTAAGCCATGCTGC
gvpGR-HindIII	CCCAAGCTTTCATTTCGTCGTACCCTCG
gvpKF-EcoRI	GGAATTCGTAGGGAGTAACACTATGAC
gvpKR-HindIII	CCCAAGCTTTTACAGCAATCGGCCTAGC
gvpF1F-EcoRI	GGAATTCATTGAGGAGTGCATGATGAG
gvpF1R-HindIII	CCCAAGCTTTCAGTCAGAGAGAGTACCAG
gvpF2F-EcoRI	GGAATTCTTGGCGATGATGCATGACG
gvpF2R-SalI	ACGCGTCGACTCATGATTCGTCAGGTTGG
gvpF3F-EcoRI	GGAATTCCAACCTGACGAATCATGAGC
gvpF3R-HindIII	CCCAAGCTTTTAGCTGGCGAAGTTATAGG
gvpNF-EcoRI	GGAATTCGCACAGGAGTATTTCCATG
gvpNR-HindIII	CCCAAGCTTTAGATAATTACTCCACTGC
gvpVF-EcoRI	GGAATTCAGTGGAGTAATTATCATGG
gvpVR-HindIII	CCCAAGCTTAACCTCAATACTTGATTCGC
gvrAF-EcoRI	GGAATTCCCAATTGGATGTAGTCGATG
gvrAR-HindIII	CCCAAGCTTCTACTCATGATTGTTTACC
gvrBF-BamHI	CGGGATCCTAACGACAGGCTACCATGTCAG
gvrBR-HindIII	CCCAAGCTTTTAGAGTATAGGTAAGG
gvrCF-EcoRI	GGAATTCACCTATACTCTAAAATGATG
gvrCR-HindIII	CCCAAGCTTCAACTACTCGCTCGGCTGTG
oREM399	GCTAATCAGGAGCGTTAATTGA
oREM400	AGCAGGAGAGCGCGTTGATA
oREM397	AAGCTTTAAGTCTAACTACTGTTGAATTC
oREM398	GTTTAGGTTACTCATCGTTTTGG
